# Hong-Hui-Xiang Alleviates Pain Hypersensitivity in a Mouse Model of Monoarthritis

**DOI:** 10.1155/2020/5626948

**Published:** 2020-12-08

**Authors:** Wei Gao, Di Wang, Xinlu Yang, Tingting Pan, Xiaoqing Chai, Zhi Zhang

**Affiliations:** ^1^Anhui Provincial Hospital, Cheeloo College of Medicine, Shandong University, Jinan, Shandong 250021, China; ^2^Department of Anesthesiology and Pain Medicine, Anhui Provincial Hospital, First Affiliated Hospital of USTC, Division of Life Sciences and Medicine, University of Science and Technology of China (USTC), Hefei 230001, Anhui, China

## Abstract

**Background:**

Hong-Hui-Xiang (HHX) is a sterilized aqueous solution extracted from *Illicium lanceolatum* A.C. Smith widely used for pain relief in China. Despite its history, it is not well understood. In the present study, we used a mouse model of arthritic knee pain to investigate the antinociceptive effects of HHX and its potential side effects on weight and respiratory function, as well as on the liver, kidney, and heart.

**Methods:**

Mice were randomly assigned to four groups: saline and HHX at three doses (1 *μ*l, 10 *μ*l, and 50 *μ*l). Each group was randomly divided to two subgroups: saline and CFA. After the first injection of HHX or saline on day 7, mechanical hyperalgesia was tested via the hind paw. Only after the tests had established that the analgesic effect had subsided was the next injection administered. A total of five injections were administered. Blood, knee joints, and other organs were collected for histopathological observation and biochemical detection.

**Objectives:**

We found that mechanical threshold of hind paw increased 2 h after of the initial injection HHX (10 *μ*l and 50 *μ*l), which lasted for at least 3 h. The analgesic effect lasted for three days after the second injection on day 8 and was approximately maintained for five days each time after the third injection. We also found a reduction in the diameter of the knee joint and suppression of synovial inflammation in response to treatment of HHX (10 *μ*l and 50 *μ*l). Meanwhile, HHX had no toxic effects on the liver, kidneys, and heart via histological and biochemical assays in all groups.

**Conclusion:**

HHX exerts antinociceptive and anti-inflammatory effects in a mouse model of arthritic knee pain. There were no obvious side effects on the liver, kidneys, or heart.

## 1. Introduction

Chronic pain, especially severe pain, can lead to a decline in one's quality of life and can have a detrimental impact on mental health [1]. Unfortunately, the currently available drugs to treat chronic pain and any comorbidity have incomplete efficacies. Opioids are widely used and are one of the most effective drugs for treating moderate to severe pain. However, long-term treatment with opioids can induce a variety of side effects, such as drug dependence and abuse, as well as overdose-related deaths [2]. Additionally, opioids exhibit poor analgesic effects in multiple neuropathic pain [3]. These adverse effects hamper the optimal clinical use of opioids in the treatment of chronic pain conditions.

Medicinal plants have been used for health and disease management in humans and other animals worldwide. In some developing countries, about 80% of the population depends on traditional medicine for the primary form of healthcare [4]. Similarly, there is an increasing interest in traditional medicine in developed countries. Hong-Hui-Xiang (HHX) is made from the root or branch of *Illicium lanceolatum* A.C. Smith, which is one kind of traditional Chinese medicinal plant of the genus *Illicium* (Illiciaceae family). In the past, research on this plant has mainly focused on its botanical and phytochemical properties [5, 6]. More than 104 compounds have been isolated from *Illicium lanceolatum* A. C. Smith. including volatiles, seco-prezizaane-type sesquiterpenes, phenylpropanoids, lignans, flavonoids, and other constituents, among which flavonoids have been found to exhibit antimicrobial, antioxidant, insecticidal, analgesic, sedative, and convulsive activities, and seco-prezizaane-type sesquiterpenes have been shown to exhibit diverse biological activities including neurotoxic and neurotrophic effects [7, 8]. Of note, HHX from *Illicium lanceolatum* has been used for conditions with chronic, severe pain, such as arthritis, for hundreds of years in China. Specifically, the relief of joint pain is an important goal in the treatment of arthritis. However, the mechanism for HHX underlying pain relief remains unknown.

Research regarding the mechanisms of HHX-mediated analgesia has been limited because there has not been an appropriate model of inflammatory and persistent pain for elucidating the molecular, cellular, and behavioral features of HHX treatment. Understanding these features is important because the toxicity, safety, side effects, and molecular targets of HHX need to be characterized if HHX is to be widely used in clinical applications. Some features of arthritis, such as inflammation, become detectable before patients experience pain symptoms. They likely prime and cause pain symptoms.

In the present study, we used a mouse model of arthritic inflammatory pain to investigate the effect of HHX on local inflammatory cell infiltration and swelling of the joints in arthritis, which may be associated with pain symptoms and with the side effects of HHX. Of note, the safety, quality, and effectiveness of traditional medicines still remain a concern [9]. In this study, changes in bodyweight gain were used as an indicator of general health status of experimental animals. Histopathological findings and serum biochemical measurement were used as sensitive indicators of toxicity and target organ injury.

## 2. Materials and Methods

### 2.1. Animals

C57BL/6J male mice were purchased from Jackson Laboratories at 8–12 weeks of age (20–30 g). All mice were housed at five mice per cage in a colony with access to food and water ad libitum and were maintained under a 12-h light/dark cycle (lights on from 7:00 a.m. to 7:00 p.m.) at a stable temperature (23°C–25°C). All animal protocols were approved by the Animal Care and Use Committee of the University of Science and Technology of China. Mice were acclimated to the housing conditions for one week prior to the experiments. A total of six mice were assigned to each experimental group.

### 2.2. Animal Model of Joint Pain

**Figure 1:**
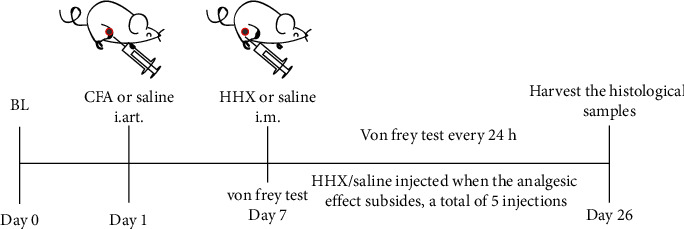
Flow chart of the study evaluating the antinociceptive effects of HHX in a mouse model of monoarthritis pain.

Complete Freund's adjuvant (CFA) with 1 mg/ml heat-killed *Mycobacterium butyricum* was commercially available from Sigma and was used in the present study. The protocol of the experiment is shown in Figure 1. As described previously [10], a mouse model of arthritic joint pain was generated via a single intraarticular injection of 10 *μ*g/10 *μ*l of CFA into the right hindlimb knee joint using an insulin syringe (BD Microfine insulin syringe, 0.3 ml; BD Medical, Oxford, UK), while the mice were briefly anesthetized with isoflurane. Saline (0.9% NaCl) was injected as a control. The day of CFA injection was designated as day 0, and the unilateral monarthritic reaction persisted until approximately day 28 [11]. Behavioral measurements of hyperalgesia were obtained at baseline and at indicated time points. All experiments were conducted in a blinded manner, and CFA/saline injections were randomly assigned.

### 2.3. Drugs

Hong-Hui-Xiang (HHX) was provided by Zhejiang Taikang Pharmaceutical Group Co., Ltd. The State Food and Drug Administration of China's approval number is Z33020931. It is included in the Drug Standard of Ministry of Public Health of the People's Republic of China—Traditional Chinese Medicine Prescription Preparation Volume No. 20. HHX is a sterile injectable solution containing ingredients from *Illicium lanceolatum* A.C. Smith prepared by soaking, filtration, concentration, filtration, ultrafiltration, dilution, sealing, sterilization, and other processes. This new ultrafiltration process is effective in removing large molecules that may be harmful to human health. The content and quality control data are as follows: total flavonoid (rutin, C_27_H_30_O_16_) is not less than 4.0 mg ml^−1^. The content of rutin from HHX in this study is 7.2 mg ml^−1^.

### 2.4. Groups and Treatments

The mice were randomly assigned to the following four groups: saline (50 *μ*l of 0.9% NaCl, i.m.), 1 *μ*l of HHX (dissolved in 50 *μ*l of saline, i.m.), 10 *μ*l of HHX (dissolved in 50 *μ*l saline, i.m.), and 50 *μ*l of HHX (i.m.). Mice within each group were then randomly assigned into two subgroups, saline or CFA, forming a total of eight groups: (1) saline-saline, (2) saline-HHX-1 *μ*l, (3) saline-HHX-10 *μ*l, (4) saline-HHX-50 *μ*l, (5) CFA-saline, (6) CFA-HHX-1 *μ*l, (7) CFA-HHX-10 *μ*l, and (8) CFA-HHX-50 *μ*l.

### 2.5. Mechanical Sensitivity

Before testing, mice were habituated to both the experimenter and to a plexiglass restrainer on an elevated platform with a mesh wire top for at least two days, for 30 min each day. After 30 min of acclimation on the elevated platform, mechanical hyperalgesia at the hind paw was tested using an electronic von Frey anesthesiometer (IITC Life Science Inc., Woodland Hills, CA, USA), as previously described [12]. Prior studies in murine models of unilateral arthritis induced by single intraarticular injections have indicated that secondary hyperalgesia develops in response to both mechanical and heat stimuli at the hind paw [13–15]. Measurements were stopped when the paw was withdrawn, and results are expressed as the mean mechanical allodynia (g) over three experiments. The baseline and indicated time points of mechanical allodynia at the ipsilateral hind paw were measured, as shown in Figure 1. At day 7, mechanical allodynia was measured, and a mouse was excluded from further analysis if its mechanical allodynia was greater than a mean of 5 g. The mean mechanical allodynia was measured at 10, 20, and 30 min, as well as at 1, 1.5, 2, 3, 4, 5, and 6 h after the first injection of HHX/saline. The mean value of mechanical allodynia was tested every 24 h and at 2, 4, and 6 h after each injection. Once the measurements indicated that the analgesic effect of HHX had subsided, a subsequent injection was performed, and mechanical allodynia was tested at 2, 4, and 6 h after this injection, until there were a total of five injections.

### 2.6. General Assessments

The knee diameter and bodyweight were measured every week, from day 0 until the end of the experiment, by two independent observers who were blinded to the treatment protocol. Additionally, the respiration rate of each mouse was measured after each injection.

### 2.7. Histological Examinations

Mice were anesthetized with sodium pentobarbital (50 mg/kg, i.p.) and were then intracardially perfused with saline and 4% (w/v) paraformaldehyde. The knee joints, including the muscle and bone, were collected and fixed in 4% (w/v) paraformaldehyde, decalcified for four weeks with 5% ethylenediaminetetraacetic acid (EDTA), were paraffin-embedded, and were then sectioned and stained with hematoxylin and eosin (H&E). Then, H&E sections were scored for synovial hypertrophy, mononuclear cell infiltration, cartilage destruction, and bone erosion by an experimenter who was blind to the experimental groups. A 0–3-point scale was used for each parameter (0 = normal, 1 = mild inflammation, 2 = moderate inflammation, and 3 = severe inflammation); the maximum possible score was 12.

Livers, kidneys, and hearts were collected and fixed in 4% (w/v) paraformaldehyde, preserved for 24 h, dehydrated through a series of graded ethanol, hyalinized with xylene, embedded in paraffin, and sectioned into 5 *μ*m-thick sections. Microsections were stained with H&E. Histological changes were observed on an Olympus CKX53 microscope.

### 2.8. Biochemical Examinations

Blood was collected for biochemical assays at the end of the experiment. The serum levels of aminotransferase (ALT), aspartate aminotransferase (AST), creatinine (Cr), urea (urea), and creatine kinase isoenzyme MB (CK-MB) were measured using a Rayto Chemray 240 Chemistry Analyzer (Rayto Life and Analytical Sciences Co., Ltd.). The values are expressed as U/L, mmol/L, or *μ*mol/L in serum.

### 2.9. Statistical Analysis

All data are presented as the mean ± standard error of the mean (SEM). Repeated measures were used for statistical analyses of behavioral nociception and knee joint diameter. One-way analyses of variance (ANOVAs) with post hoc Tukey's tests were applied for statistical analyses of weights, respiratory rates, knee joint scores, and biochemical parameters. *P* < 0.05 was considered statistically significant.

## 3. Results

### 3.1. Dose-Dependent and Time-Dependent Antinociceptive Effects of HHX

**Figure 2:**
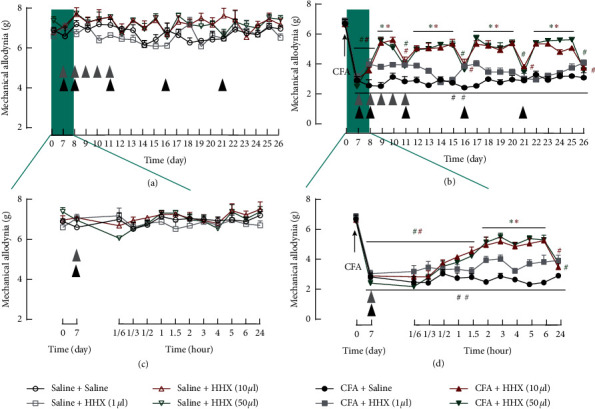
The time course of the effects of HHX (1, 10, and 50 *μ*l i.m.) on pain thresholds after saline (A and C) or CFA articular injection (B and D). (a) HHX at doses of 1, 10, and 50 *μ*l did not change the mechanical hypersensitivity after saline articular injection (time × group interaction, *F*_(20, 200)_ = 1.264, *P*=0.2072; *F*_(20, 200)_ = 0.8155, *P*=0.6932; *F*_(20, 200)_ = 0.8660, *P*=0.6303). (c) Rectangular box denoted postdrug administration behavioral assessment at different points in time after the first injection. Mechanical allogynia decreased moderately 10 min after the first injection of HHX (50 *μ*l). (b) HHX at doses of 10 *μ*l and 50 *μ*l changed the mechanical hypersensitivity after CFA articular injection (time × group interaction, *F*_(20, 200)_ = 7.859, *P* < 0.0001; *F*_(20, 200)_ = 11.84, *P* < 0.0001). However, HHX at a dose of 1 *μ*l had no antinociceptive effects (time × group interaction, *F*_(20, 200)_ = 1.336, *P*=0.1596). (d) The rectangular box denotes postdrug administration behavioral assessment at different points in time after the first injection. The first injection of HHX provided rapid antinociception at the hind paw at 2 h after the injection and lasted for approximately 4 h. The time of the injection of HHX (1 *μ*l) is shown as gray arrows and HHX (10 and 50 *μ*l) shown as black arrows. ^*∗*^*P* < 0.05 versus CFA + saline. ^#^*P* < 0.05 versus baseline.

According to the instructions provided with HXX by the manufacturer, intramuscular drug with a dose of 1-2 ml at pain spots, rather than systemic route, is recommended. Thus, in this study, C57BL/6 mice received intraarticular injections of CFA (10 *μ*g/10 *μ*l) or saline (10 *μ*l) into the right hindlimb knee joint. The drug dose of 10 *μ*l HHX was calculated for mice based on the conversion of human drug dosage according to the book Laboratory Animal Science (ISBN: 9787565506734). A low-dose group (HHX 1 *μ*l) and a high-dose group (HHX 50 *μ*l) was established to assess any dose-dependent antinociceptive or side effects of HHX. Intraarticular injection of CFA caused significant hyperalgesia in the ipsilateral hind paw. HHX at doses of 10 *μ*l and 50 *μ*l had significant antinociceptive effects (*P* < 0.001) on CFA-induced secondary hyperalgesia; however, HHX at a dose of 1 *μ*l had no antinociceptive effects. The time course of antinociceptive effects is shown in Figure 2. At the acute phase, the antinociceptive effects of HHX were brief. The initial injection of HHX (10 or 50 *μ*l) at day 7 provided rapid antinociception at the hind paw at 2 h after the injection and lasted for approximately 4 h, after which the effect subsided by 24 h. The second injection was administrated at day 8, the effects of which lasted until day 11. Subsequent injections were administrated at days 11, 16, and 21. Generally, the analgesic effects of each of the latter three injections lasted for five days.

### 3.2. Histological and General Examinations

**Figure 3:**
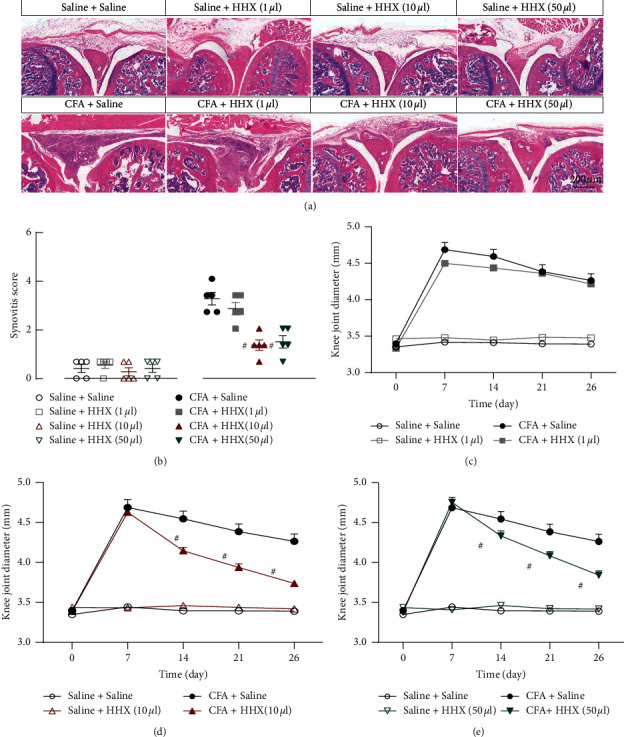
The anti-inflammatory effect of HHX in a mouse model of monoarthritis. (a) Histological changes of synovitis were observed in H&E-stained sections. Each knee joint shown was a representative for a group of mice; scale bars represents 200 *μ*m. (b) The synovitis scores for each group from the H&E-stained sections. Synovitis scores in CFA increased significantly (*t*_8_ = 9.391, *P* < 0.0001) and decreased in HHX at doses of 10 *μ*l and 50 *μ*l (*t*_8_ = 5.715, *P*=0.0004; *t*_8_ = 4.914, *P*=0.0012), but not at the dose of 1 *μ*l (*t*_8_ = 1.134, *P*=0.2897) at day 26. ((c)–(e)) The effect of HHX (1, 10, and 50 *μ*l) on knee joint thickness. CFA exhibited a significant increase in knee joint thickness (time × group interaction, *F*_(4, 40)_ = 98.74, *P* < 0.0001). Time course of HHX at doses of 10 *μ*l and 50 *μ*l decreased the knee joint diameter (time × group interaction, *F*_(4, 40)_ = 20.52, *P* < 0.0001; *F*_(4, 40)_ = 14.45, *P* < 0.0001). However, HHX at doses of 1 *μ*l did not decrease the knee joint diameter (time × group interaction, *F*_(4, 40)_ = 1.597, *P*=0.1992). ^#^*P* < 0.05 versus CFA + saline.

Mice were followed up for 12 or 26 days. The extent of articular inflammation over the course of the disease was assessed via measurement of joint diameter using calipers and assessment of histological changes. Mice treated with CFA exhibited a significant increase in knee joint thickness and the total pathological score (based on synovial hypertrophy, mononuclear cell infiltration, cartilage destruction, and bone erosion scores) throughout the measurement period, as compared to these parameters in the sham joint (*P* < 0.0001) (Figure 3). HHX at doses of 10 *μ*l and 50 *μ*l decreased the knee joint diameter on days 14, 21, and 26 and relieved synovial inflammation (i.e., synovial hyperplasia and inflammatory cell infiltration were reduced) on day 26. HHX at a dose of 10 *μ*l and 50 *μ*l induced significant inhibitory effects on CFA-induced unilateral arthritis in mice, which was not observed at a dose of 1 *μ*l.

### 3.3. Adverse Effects and Safety

**Figure 4:**
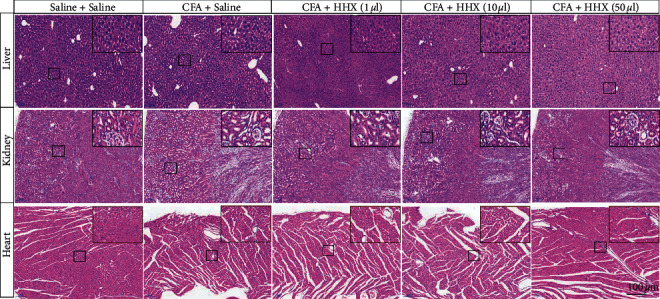
The histological structural of the liver, kidney, and heart was observed in H&E-stained sections. There were no significant differences between the different groups.

**Figure 5:**
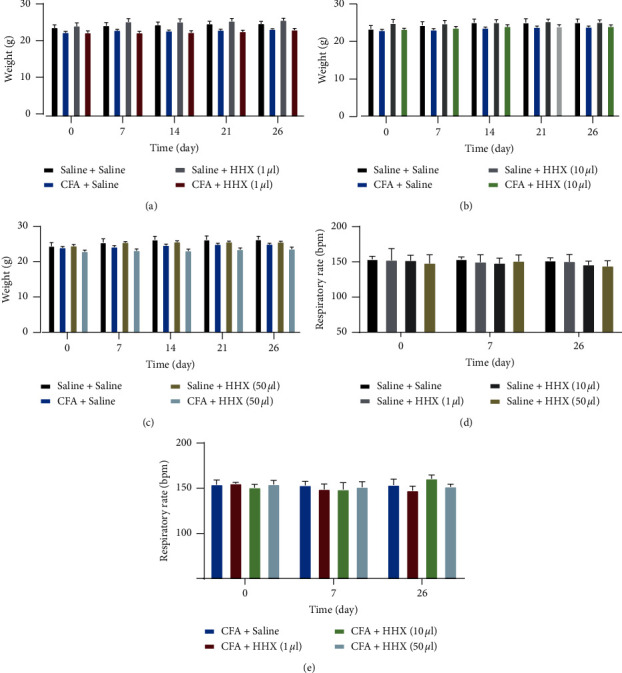
Bodyweight over time between groups ((a), (b), and (c)). There were no significant differences in the bodyweight after the treatment of HHX at doses of 1 *μ*l, 10 *μ*l, or 50 *μ*l (column factor, *F*_(3, 16)_ = 0.4253, *P*=0.7375; *F*_(3, 16)_ = 1.077, *P*=0.3870; *F*_(3, 16)_ = 1.690, *P*=0.2091). The effect of HHX on the respiratory rate ((d) and (e)). There were no significant differences in respiratory rate across the different saline subgroups or CFA subgroups (time × group interaction, *F*_(6, 40)_ = 0.5040, *P*=0.8016; *F*_(6, 40)_ = 1.903, *P*=0.1042).

**Figure 6:**
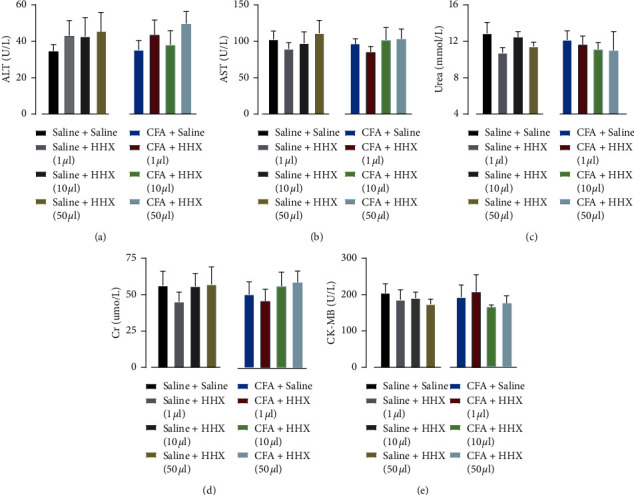
The effect of HHX on blood biochemical index. There were no significant differences of serum levels of ALT (treatment between columns, *F*_(7, 39)_ = 0.3886, *P*=0.9033), AST (treatment between columns, *F*_(7, 39)_ = 0.3444, *P*=0.9282), Cr (treatment between columns, *F*_(7, 39)_ = 0.3247, *P*=0.9382), urea (treatment between columns, *F*_(7, 39)_ = 0.4097, *P*=0.8905), and CK-MB (treatment between columns, *F*_(7, 39)_ = 0.3788, *P*=0.9091) across the different groups at day 26.

There were no significant differences in the histological structural features of the livers, kidneys, or hearts between the different groups (Figure 4). The morphology of liver cells was regular, the liver cords were arranged neatly, the liver lobule structure was integrated, and no steatosis or inflammatory cell infiltration was observed in any of the groups. There were no obvious abnormalities in the morphologies or structures of glomeruli, renal tubules, or the renal interstitium. Myocardial cells exhibited an orderly arrangement and integrated structure, and hypertrophy was not observed in the atria or ventricular muscles. Furthermore, inflammatory cell infiltration was not observed. HHX had no significant effect on bodyweight or respiratory rate (Figure 5). Additionally, serum levels of ALT, AST, Cr, urea, and CK-MB did not change between the different groups (Figure 6).

## 4. Discussion

HHX is widely prescribed for analgesia in China. Although the mechanism of HHX on pain is unknown, HHX is known to exhibit sufficient and effective analgesic action in clinical practice, and adverse events are rarely reported. In the present study, we found that HHX relieved joint pain at doses of 10 *μ*l and 50 *μ*l, with a short duration of action in the acute phase. Following the third administration of HHX, the analgesic effects lasted for approximately five days. HHX has similar analgesic effects as those of opioids, but do not exhibit any opioid-related adverse effects. Although opioids represent the main analgesics for many types of acute pain, the benefits of opioids in chronic pain have been contested since the long-term use of opioids can lead to serious adverse effects such as drug dependence, addiction, and desensitization [16, 17]. Furthermore, some previous studies have shown that opioids do not exert significant pain relief or pain-related functional improvements in the treatment of moderate to severe chronic back pain, hip pain, or knee pain compared to that of nonopioid medications given over the course of 12 months. Additionally, opioids cause more treatment-related side effects compared to those of nonopioid medications [18]. We found that efficacious doses of HHX did not need to be increased during the treatment period, and that HHX achieved a consistent analgesic effect over a long treatment course. Furthermore, physical dependence and tolerance were not found after the use of HHX.

In the present study, no heart, liver, or kidney toxicity was observed, even at high doses of HHX. The liver and kidneys are important organs, which are responsible for the metabolism, detoxification, and excretion of xenobiotics. These organs are susceptible to damage by external substances [19]. Their functionality can be measured by serum biochemical analysis. Some traditional medicines can induce cardiotoxicity. The serum concentration of CK-MB, which are diagnostic markers of myocardial damage, can indicate the degree of cell membrane integrity. Our data showed that the histological structural features and biochemical parameters of the livers, kidneys, and heart did not change after administration of HHX. HHX also did not affect bodyweight gain or the respiratory rate, both of which indicate the general health status of mice. These findings are consistent with the manufacturer's instruction of HHX. Thus, the current animal models are probably suitable for investigation of more different functions of HHX.

According to findings from previous studies [20–22], we propose that four potential ingredients from HHX—shikimic acid, pseudoshikimic acid, quercetin, and taxifolin—are likely involved and may underlie the analgesic or anti-inflammatory effects of HHX. Shikimic acid has been reported to have anti-inflammatory and analgesic actions through inhibition of ERK 1/2 and p38 MAPKs [21], and quercetin has analgesic effects that are probably related to the regulation of GABA_A_, GABA_B_, tachykinin, and 5-HT receptors and to the endogenous release of glucocorticoids [22]. In addition, quercetin alleviates diabetic neuropathic pain by inhibiting mTOR/p70S6K pathway-mediated changes of synaptic morphology and synaptic protein levels in spinal dorsal horn neurons. It also inhibits the peripheral and central sensitization of bone cancer pain by inhibiting the PAR2/TRPV1 signaling pathway [23, 24]. Taxifolin inhibits the leukocyte infiltration and the expressions of COX-2 and iNOS in the brain with cerebral ischemic reperfusion injury, also prevents the expression of Mac-1 and ICAM-1, and inhibits the activity of NF-*κ*B [25]. Based on these reports, we speculate that HHX might alleviate pain in multiple ways, e.g., by reducing the local inflammatory response and by changing synaptic morphology. Given HHX can affect multiple pain-related signaling pathways like a cocktail, it can produce more desirable antinociceptive effects in complicated pain conditions than those agents that target one pathway. In the present study, HHX alleviated synovial inflammation, including synovial hyperplasia and inflammatory cell infiltration, and also inhibited joint swelling as indicated pathological examination. This may be why HHX exerts analgesic effects.

For antinociceptive purposes, although few reports of target-organ toxicity or side effects have been documented in academic and clinical reports, the adverse effects cannot be disregarded and should been investigated in the future. HHX is made from *Illicium lanceolatum* A.C. Smith. The roots and fruits of *Illicium lanceolatum* have visible central excitatory and peripheral muscarinic effects. An overdose of *Illicium lanceolatum* first stimulates and then inhibits the human brain and spinal cord, eventually leading to respiratory and circulatory failure, even death [26, 27]. This indicates that *Illicium lanceolatum* could have adverse effects in some conditions. In addition, numerus compounds, which may cause adverse effects, are involved in the extract of *Illicium lanceolatum*, which is not absolutely excluded in HHX extraction. For example, administration of high dose (500 mg/kg, p.o.) of *Illicium lanceolatum* ethyl extract veranisatins produces convulsions and lethal toxicity in mice [28, 29]. Reports have shown that the *Illicium lanceolatum* extracts, anisatin and neoanisatin, are the toxic components [30–32]. The mechanism underlying the toxic effect may involve anisatin acting as a potent noncompetitive antagonist in GABA-dependent neurons [33, 34]. Although HHX is a water extract from *Illicium lanceolatum* A.C. Smith, which mainly contains flavonoids but not harmful seco-prezizaane-type sesquiterpene compounds, we cannot rule out other toxic compounds in the HHX injection. These potential issues may remind researchers to investigate HHX further. Their findings may promote wider clinical application of HHX for pain relief in arthritis and other conditions.

Taken together, we found that HHX exhibited a safe and effective analgesic effect in murine unilateral arthritis induced by CFA and that the long time course of this efficacy holds promise for clinical applications. However, further studies are needed to elucidate the mechanisms of HHX in exerting anti-inflammatory and analgesic actions in CFA-mediated arthritic mice.

## 5. Conclusions

Mouse monoarthritis induced by CFA is an appropriate model for examining the pharmic effects of the extract of *Illicium lanceolatum* A.C. Smith. The parenteral solution exerts an anti-inflammatory effect and alleviates knee joint pain in a non-dose-dependent manner. It showed no opioid side effects or any cardiac, hepatic, or renal toxicity when administered at the therapeutic dose.

## Data Availability

The data used to support the findings of this study are available from the corresponding author upon request.
